# Improving the Self-Healing of Cementitious Materials with a Hydrogel System

**DOI:** 10.3390/gels8050278

**Published:** 2022-04-29

**Authors:** Hao Wang, Mohammad Habibi, Riadh Marzouki, Ali Majdi, Morteza Shariati, Nebojsa Denic, Aleksandar Zakić, Majid Khorami, Mohamed Amine Khadimallah, Ahmed Abdel Khalek Ebid

**Affiliations:** 1 School of Civil Engineering, Chongqing Vocational Institute of Engineering, Chongqing 402260, China; whdyx147896325@163.com; 2 Department of Civil Engineering, Calut Company Holding, Melbourne 3800, Australia; mohammad@calut.com.au; 3 Chemistry Department, College of Science, King Khalid University, Abha 61413, Saudi Arabia; rmarzouki@kku.edu.sa; 4 Department of Building and Construction Techniques, Al-Mustaqbal University College, Babylon 51001, Iraq; alimajdi@mustaqbal-college.edu.iq; 5 Department of Civil Engineering Discipline, School of Engineering, Monash University, Melbourne 3800, Australia; 6 Faculty of Sciences and Mathematics, University of Priština, 38220 Kosovska Mitrovica, Serbia; nebojsa.denic@pr.ac.rs; 7 Faculty of Mathematics and Computer Science, ALFA BK University, 11070 Belgrade, Serbia; aleksandar.zakic@alfa.edu.rs; 8 Facultad de Arquitectura y Urbanismo, Universidad UTE, Calle Rumipamba S/N y Bourgeois, Quito 170147, Ecuador; majid.khorami@ute.edu.ec; 9 Department of Civil Engineering, College of Engineering, Prince Sattam bin Abdulaziz University, Al-Kharj 16273, Saudi Arabia; m.khadimallah@psau.edu.sa; 10 Laboratory of Systems and Applied Mechanics, Polytechnic School of Tunisia, University of Carthage, Tunis 1054, Tunisia; 11 Structural Engineering and Construction Management, Faculty of Engineering, Future University, Cairo 11835, Egypt; ahmed.abdelkhaleq@fue.edu.eg

**Keywords:** self-healing, cement, hydrogel, water glass, nanosilica

## Abstract

Despite cement’s superior performance and inexpensive cost compared to other industrial materials, crack development remains a persistent problem in concrete. Given the comparatively low tensile strength, when cracks emerge, a pathway is created for gas and water to enter the cementitious matrix, resulting in steel reinforcement corrosion which compromises the durability of concrete. Superabsorbent hydrogels have been developed as a novel material for enhancing the characteristics of cementitious materials in which they have been demonstrated to decrease autogenous shrinkage and encourage self-healing. This study will detail the design and application of polyelectrolyte hydrogel particles as internal curing agents in concrete and provide new findings on relevant hydrogel–ion interactions. When hydrogel particles are mixed into concrete, they generate their stored water to fuel the curing reaction that results in less cracking and shrinkage, thereby prolonging the service life of the concrete. The interaction of hydrogels with cementitious materials is addressed in this study; the effect of hydrogels on the characteristics and self-healing of cementitious materials was also studied. Incorporating hydrogel particles into cement decreased mixture shrinkage while increasing the production of particular inorganic phases within the vacuum region formerly supplied by the swollen particle. In addition, considering the control paste, cement pastes containing hydrogels exhibited less autogenous shrinkage. The influence of hydrogels on autogenous shrinkage was found to be chemically dependent; the hydrogel with a delayed desorption rate displayed significantly low shrinkage in cement paste.

## 1. Introduction

The development of cracks endangers concrete’s durability and results in reinforcement corrosion because it creates a pathway for dangerous particles dissolved in fluids and gases [1,2]. The increasing demand for internal and active therapy, or self-healing materials, has led to increased research focusing on reversing damage development during the previous decade [3]. Self-healing materials have the potential to reverse damage formation, extending the lifetime and dependability of the concrete [4]. Indeed, concrete has a natural capacity to heal damage to a certain level. Autogenous healing refers to concrete’s natural and inherent capacity to self-heal microscopic fissures caused by its composition. Autogenous healing occurs in cracks because of the hydration of un-hydrated cement particles and calcium carbonate precipitation [5].

**Figure 1:**
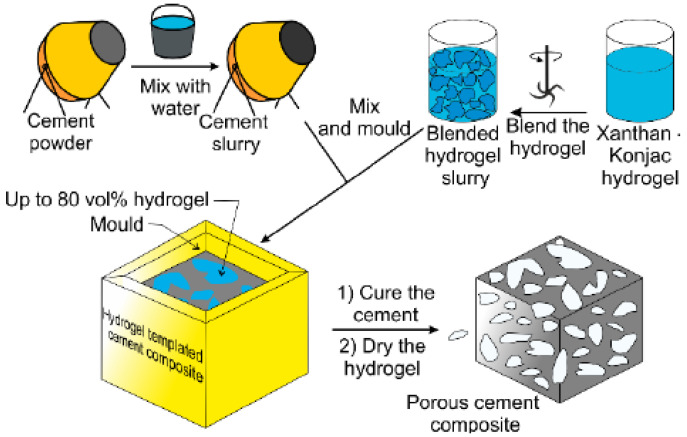
Methodology of production of porous cement through a hydrogel bead slurry.

**Figure 2:**
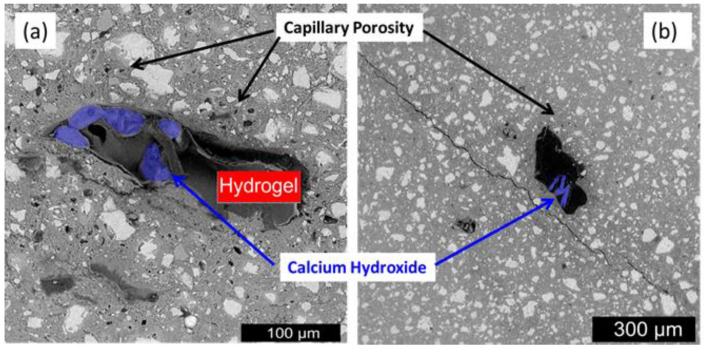
SEM micrographs of 7-day cured, cross-sectioned, and polished cement paste samples containing calcium hydroxide formations within the void space remaining from dehydrated (**a**) 17% AA and (**b**) 33% AA SAP hydrogel particles.

**Figure 3:**
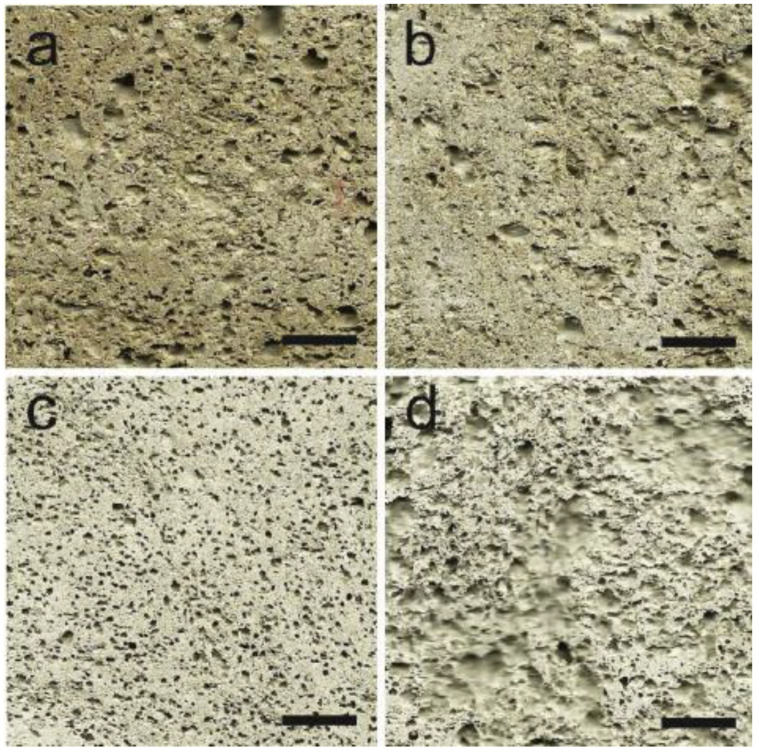
Photographs of porous cement composites: (**a**) Photographs of porous cement composites 10%, (**b**) 25%, (**c**) 50%, (**d**) 70% hydrogel.

Water permeability decreases in cracked concrete samples because of the autogenous crack healing [6]. Figure 1 depicts how hydrogel is made by first hydrating the gelling agent in water, then setting the gel and mixing or mincing it into a slurry of hydrogel beads. The sample was air-dried after the cement was cured to generate a porous cement composite [7,8]. It should be noted that the hydrogel bead size determines the size of the holes in the final porous composite. Figure 2 shows the SEM micrographs of 7-day cured, cross-sectioned, and polished cement paste samples with calcium hydroxide formations within the void space remaining from dehydrated (a) 17% AA and (b) 33% AA SAP hydrogel particles. Figure 3a–c show the photographs of porous cement composites after 50 vol% hydrogel was applied.

Hydrogels may absorb a large volume of fluid from their surroundings and keep the liquid inside their structure without disintegrating. They could be used for self-healing because their swelling activity can prevent invading fluids from entering the cracks [9,10,11,12]. Water is more available for healing by absorbing fluids from the surrounding environment during crack development. Furthermore, hydrogels are anticipated as an encapsulating medium for biological and chemical substances, such as bacteria, as a healing agent in cementitious material self-healing uses [13,14,15,16,17]. Hydrogels are three-dimensional cross-linked hydrophilic polymers. Hydrophilic groups on polymer chains, such as hydroxyl, carboxyl, and amides, may absorb and retain their weight in fluids without changing their original structures, even under pressure [18,19]. 

Hydrogels’ exceptional qualities are now the primary reason for their widespread usage in the petroleum sector [20,21,22], agriculture [23], building industry [24,25], hygiene products [26], and materials for controlled release devices [27]. Hydrogels utilized in cementitious materials are usually made of a copolymer of acrylate salt–acrylamide or a cross-linked polymer of acrylate salt [28]. These hydrogels are polyelectrolytes, and their behavior is affected by the pH and ionic content of the surrounding environment [24,28]. Autogenous shrinkage-induced cracking is a significant issue in high-performance concrete (HPC) with a low water/cement (*w/c*) ratio [29,30,31]. The internal relative humidity eventually declined (self-desiccation) in HPC with sustained hydration and water absorption, and capillary tension developed in the menisci within the pores.

**Figure 4:**
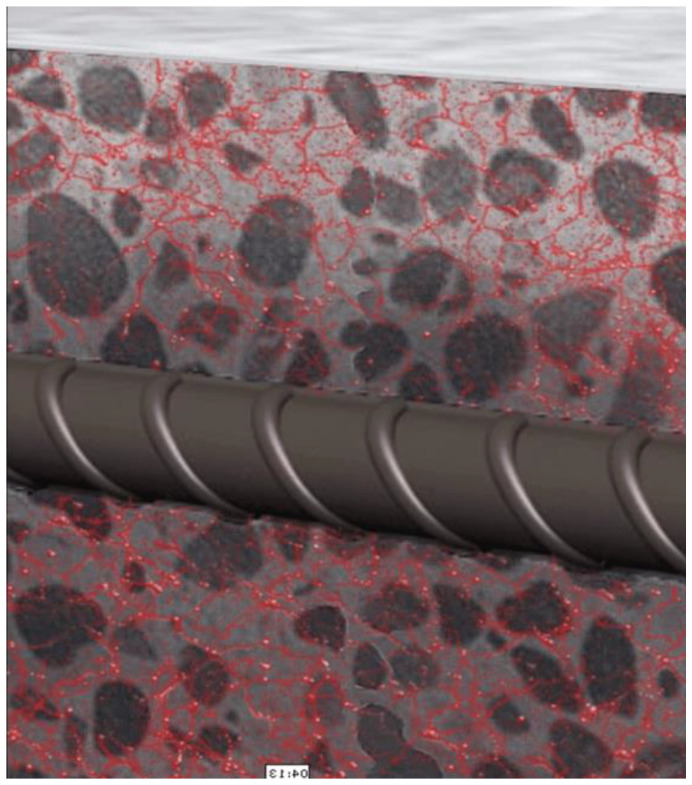
Concrete Hydrogels.

Regarding the material limitation as aggregates, autogenous shrinkage creates tensile stress resulting in crack development [32,33,34]. When cracks occur, the transfer of harmful substances into the microstructure increases, resulting in a rise in degradation processes, such as reinforcing steel corrosion and physical–chemical attacks [1,35]. Internal curing is an efficient method for reducing autogenous shrinkage in cementitious materials [29]. Water is discharged from a reservoir into the cementitious matrix during the internal curing to maintain internal relative humidity in hydration [29,36]. Hydrogels have been shown to enhance freeze–thaw resistance [37], hydration [38,39], resistance to chloride penetration [40], and stimulate self-healing in cementitious materials besides lowering autogenous shrinkage. Nevertheless, in the presence of hydrogels, compressive strength has been found to be diminished owing mostly to macrovoid formation [41] (Figure 4). The efficiency of a hydrogel is largely determined by the dose of the hydrogel employed in the cementitious matrix [42], the chemical properties of the hydrogel [29], the particle size of the hydrogel [43], and the water/binder ratio of the mixture [44]. The chemical and physical properties of hydrogels and the mix design of cementitious materials are elements that directly study the impact of hydrogels on the cementitious materials’ behavior [45]. Because of the cement hydration increment, the free water in the cementitious matrix is steadily reduced over time, leading to a decline in relative humidity [46,47]. Therefore, many capillary pores are made in the solidified cement paste, a saturation of water in the capillary pores decreases, and water menisci are formed in the capillary pores [48,49,50]. When the saturation of capillary pores changes from saturated to unsaturated, the concave inner surface of the pore experiences internal stress. Capillary tension pushes the solid surface of the pore, which causes the material to shrink overall [51,52,53,54]. Water is internally transferred from a water reservoir within the hydrogels into the cementitious matrix to achieve high RH over time in the internal curing cycle employing hydrogels, which decreases autogenous shrinkage in the material [55,56,57,58] and the beginning of cracking [59,60] (Figure 4). The efficiency of a hydrogel is largely determined by the dose of the hydrogel employed in the cementitious matrix [60,61,62,63], the chemical properties of the hydrogel [64], and the particle size of the hydrogel, and the water/binder ratio of the mixture [65,66,67].

In 2015, 92 million metric tons of concrete were manufactured in the US, providing $10.6 billion to state revenues [45]. As a result, concrete is a vast global sector with enormous potential for incorporating a diverse range of material science solutions that improve the function and sustainability of concrete materials. This material advancement lowers the concrete pavement demand and infrastructure replacement and repair over time, which bring additional economic and environmental benefits. HPC is a more sophisticated alternative to traditional concrete that has improved strength and durability due to smaller porosity and lower carbon dioxide emissions [68,69,70,71,72,73]. In contrast to the normal compressive values of (30–40) MPa, HPC may readily produce compressive strengths of 110 MPa. HPC is very resistant to corrosive fluid intrusion and because of its low porosity and unconnected pore network make it exceedingly durable even in severe conditions. HPC has strong fire resistance because of its decreased permeability, but still, the narrower pore network might produce explosive spalling at very high temperatures. Spalling could be reduced by adding soft polymers, such as crushed rubber, with a greater elastic modulus than concrete. HPC has an overabundance of Portland cement compared to free water, which causes self-desiccation. High inward Laplace pressures arise in HPC mixes when water is absorbed and drained from the smallest holes in the hydrated cement network. Extra water could be given from external sources throughout the curing procedure to prevent self-desiccation and counterbalance part of the early-age shrinkage. Nevertheless, because of the thick microstructure and limited permeability of HPC, external water has a tough time penetrating deep into the hard cement paste. 

**Figure 5:**
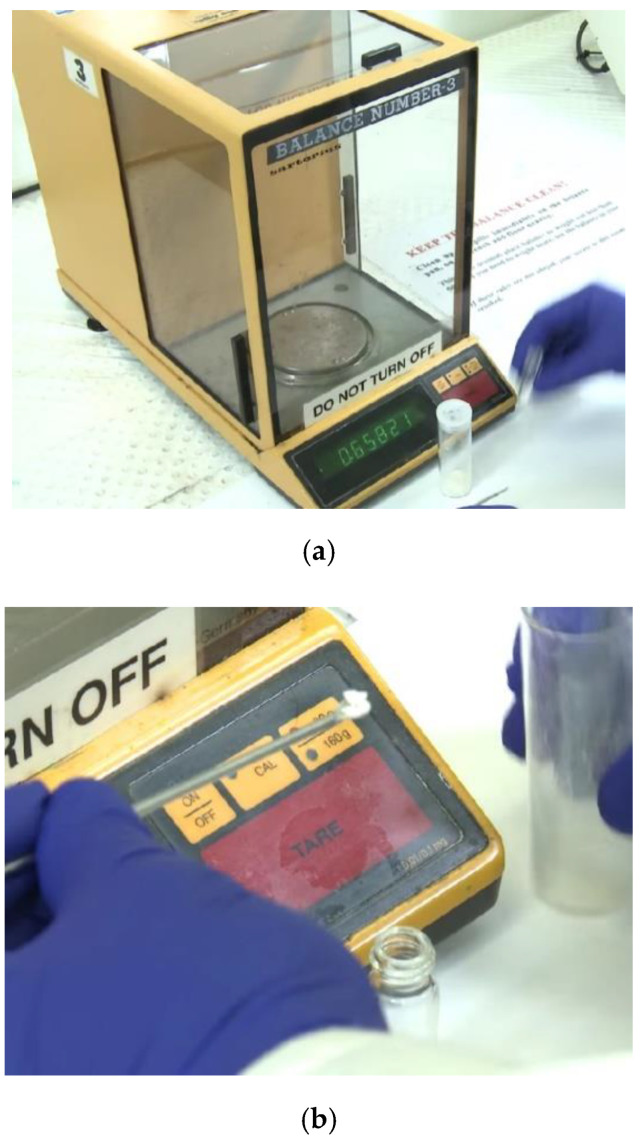

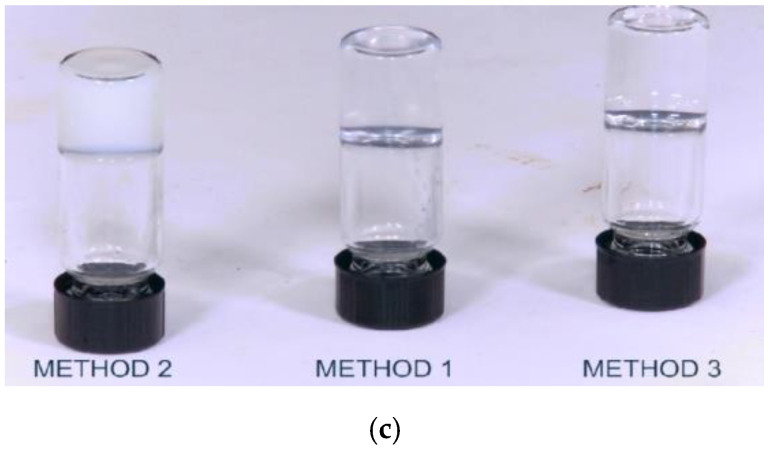
The method to prepare self-assembled hydrogel. (**a**) Weighing machine for angelator, (**b**) Usage of 20 mL of gelato into 2 mL solvent to make 1% gel, and (**c**) Adding dilute to sodium hydroxide and sonic 8.

Internal curing procedures provide a solution to the HPC self-desiccation issue. The use of covalently cross-linked superabsorbent polymer (SAP) hydrogels has recently been explored as a technique for internal curing. These hydrogels may absorb and retain their dry weight in fluid many times. The capability to adjust the mechanical response, swelling response, form, and hydrogels’ size may result in hydrogels that provide many advantages to concrete. Water and other nanoscale solutes are found to diffuse into the internal polymer network of hydrogels when submerged in aqueous solutions. There is an extra pushing factor for water and counter ion diffusion into charged polymer networks. The system is like a Donnan membrane for monovalent counterions in solution: the driving force for gel swelling may be recognized as net osmotic pressure or swelling pressure across a semipermeable membrane. Since the polymer network is charged and electroneutrality should be maintained, the concentration of free counter ions inside the hydrogel is greater than what would be predicted based only on ionic concentration. This polymer network produces a hydrogel sample with a thick polymer “skin” on the outside and a hollow core. When polymer chains are exposed to charge surfactants, they can self-assemble into ionically cross-linked gels [74]. Figure 5 shows the method for preparing self-assembled hydrogel.

### 1.1. Effect of Hydrogel Chemistry on Cement Paste

The microstructure experiment on cement pastes, including hydrogels, yielded surprising and positive findings. Since most acrylic acid hydrogels have adversely charged groups, it was hypothesized that these hydrogels would absorb more calcium ions. Considering the opposite outcomes, it appears that the availability of adequate water is more crucial than the formation of calcium-rich regions inside the hydrating cement slurry [75]. It was revealed that 17 and 33 percent of AA hydrogels did not exhibit the considerable reduction caused by the ions exhibited by the 67 and 83 percent AA hydrogels. Because of ionic interactions, the majority of acrylic acid hydrogels absorb quickly and hence cannot give adequate water to the production of hydrated products inside the voids. If hydrogels can reduce porosity, it implies that the introduction of hydrogels would not have a detrimental effect on the compressive strength of concrete. Moreover, this research clearly implies that the chemistry of hydrogels may be modified to have a variety of favorable impacts on concrete. If hydrogels could be designed to fill vacant space with the hydrated product, it may be possible to counterbalance any compressive strength loss induced by the inclusion of the hydrogels.

### 1.2. Objectives and Problem Statements

Recently, hydrogels have attracted more interest as an internal curing and self-healing agent. This work aims to determine how the chemical compositions of hydrogels affect their behavior in cementitious materials to know the impacts of hydrogels on the characteristics and microstructure of cementitious materials, and to understand their behavior in cementitious materials, particularly absorption and desorption. Regardless of the good chemical interaction between the pore solution and hydrogel, this study attempts to find the parameters influencing hydrogel absorption in cement mixes.

## 2. Materials

**Figure 6:**
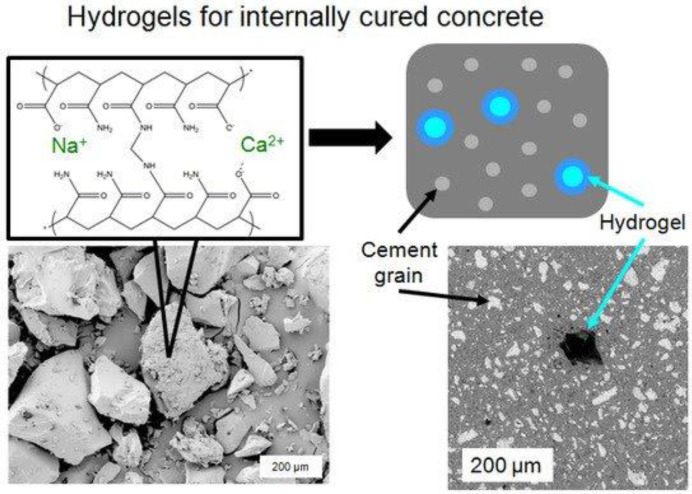
Hydrogel in cured concrete.

Other additives could be put into hydrogels to improve the cementitious healing properties. In this work, two distinct nanosilica particles (NSi) and water glass materials were integrated into hydrogels (WG). Figure 6 shows the hydrogel in cured concrete.

### 2.1. Nanosilica Particles (NSi)

Silicon dioxide nanoparticles (silica nanoparticles or nanosilica) are a type of nano-reinforcement and may be considered to be a smaller, manufactured version of silica fume. Nanosilica is available in both solid and colloidal forms; however, the colloidal form is preferable owing to agglomeration in the solid form [76]. The integration of nanosilica into the hydrogel was driven by its positive impact on cementitious materials’ hydration, mechanical characteristics, and microstructure. It is one of the most prominent admixtures in the concrete industry due to its high pozzolanic activity, compact size, and void filling capabilities. Numerous research and trials were conducted with varying concentrations of nanosilica ranging from 1% to 4% [77]. The results reveal improved mechanical characteristics and a decrease in pore volume. Because it functions as an activator to improve the pozzolanic process, adding a small quantity of nanosilica results in a considerable increase in compressive strength. According to [77], while the fineness of nanosilica increases the early strength of concrete, the ultimate strength created with coarser nanosilica is comparatively higher. In both cases, the optimal nanosilica dose was between 1.0 and 1.5 percent. [78] has reported an increase in flexural strength of concrete owing to the addition of a small quantity of nanosilica, indicating the creation of additional C-S-H gel. Likewise, the combined action of nanosilica and steel fibers significantly increases flexural strength. The inclusion of 1.5 percent nanosilica boosts the flexural strength of HPC by roughly 15%. Tensile strength and elasticity modulus were enhanced with nanosilica (with the latter ranging between 24.00 and 28.70 GPa with nanosilica content) and 13.80 and 14.53 GPa without. Nevertheless, due to the obvious large specific area of nanosilica that absorbed the water from the concrete mixes, the use of nanosilica reduces workability. The addition of nanosilica reduces both the initial and final setting times, and the disparity between the initial and final setting times decreased as the nanosilica concentration increases.

Because of the small size and huge surface area of nanosilica, hydration occurs at a quicker rate. Concrete’s major durability qualities include water absorption and permeability, chloride permeability, and so on. It has been observed that adding 2% nanosilica enhances water permeability resistance by improving the ITZ zone. Furthermore, the inclusion of nanosilica lowers water absorption indicating fewer capillary holes and greater permeability resistance. However, increasing nanosilica concentration improves the water penetration depth due to nanosilica agglomeration. Furthermore, nanosilica outperforms microsilica in terms of permeability. These favorable benefits are attributable to three main material properties. Because of its small particle size, nanosilica functions as a filler for smaller holes. Second, since nanosilica is a pozzolanic material—it can generate hydration products at a later age. Furthermore, the increased surface area acts as the main location for Portland precipitation, accelerating the hydration process. In the presence of water, the reaction between sodium silicate and calcium hydroxide is described as [79]:

(1) $${\mathrm{SiO}}_{2}+\mathrm{Ca}(\mathrm{OH}{)}_{2}+H_{2}O\;\to \;\mathrm{CaO}\cdot {\mathrm{SiO}}_{2}\cdot \left(n+1\right)H_{2}O$$

### 2.2. Water Glass

Sodium silicate is an established, low-cost industrial product with a wide range of uses. Sodium silicate has the chemical formula Na^2^O, SiO_2_; the modulus is the mole fraction ratio of SiO_2_ to Na^2^O in the molecular formula. Sodium silicate has a modulus that spans from 1 to 3.5. Sodium silicate is already used in a variety of cementitious products. It is employed as an alkali-activator in alkali-activated cement, for example. It is used as a setting accelerator in concrete and as a silicate mineral to increase waterproofing and durability. The reaction between sodium silicate and calcium hydroxide is stated in the presence of water as [80]:

(2) $${\mathrm{Na}}_{2}{\;\mathrm{SiO}}_{2}+\mathrm{Ca}(\mathrm{OH}{)}_{2}+{\;H}_{2}O\to \;x\left(\mathrm{CaO}\cdot {\mathrm{SiO}}_{2}\right)H_{2}O+{\mathrm{Na}}_{2}O$$

### 2.3. Hydrogels

In this work, poly (sodium acrylate-co-acrylamide) copolymers with various chemical compositions were investigated, and their compositions are presented in Table 1. All ingredients used in the hydrogel synthesis were bought from Sigma-Aldrich (Burlington, MA, USA) in the quantities specified. In this research, hydrogels are classified into two types. The first group, designated by the letters H-2, H-3, and H-a, consists of acrylic-based polymer hydrogels that do not contain silica components. In the second category, healing elements were integrated into hydrogels numbered 4 to 10.

#### 2.3.1. H-a Hydrogel

**Figure 7:**
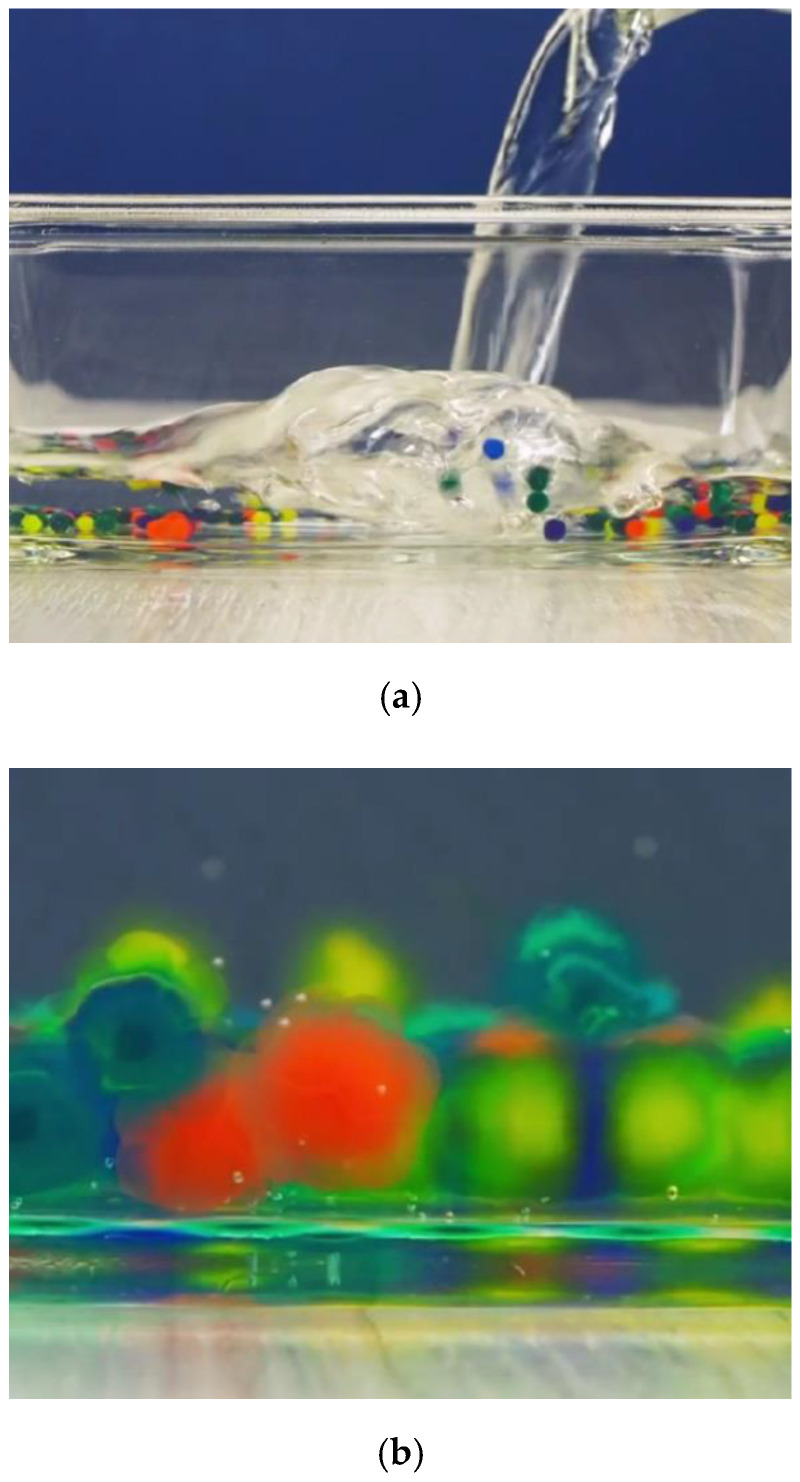
Hydrogel granolas increase in size in the presence of water (**a**) before and (**b**) after presences of water.

**Figure 8:**
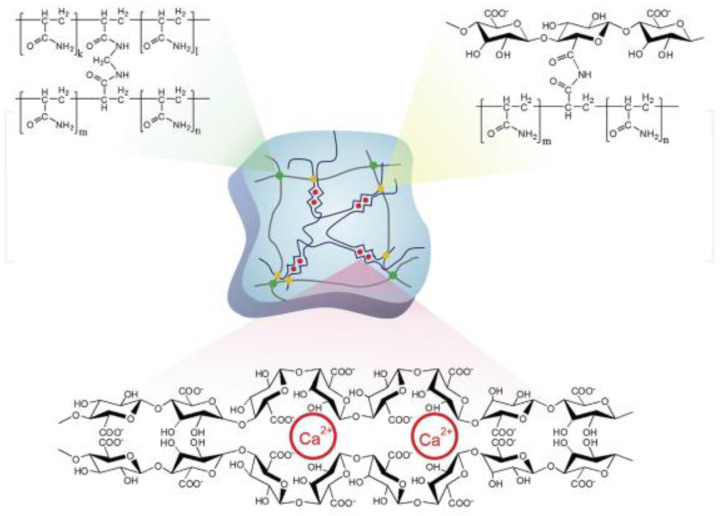
In situ hydrogel formation using chemical cross-linking and ionic interaction between alginate and calcium ions.

**Figure 9:**
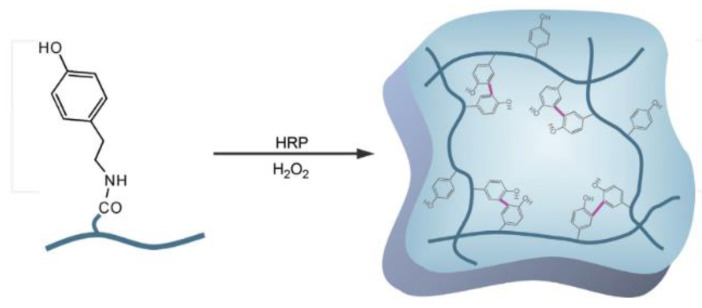
In situ hydrogel-forming by the interaction of H_2_O_2_ and horseradish peroxidase (HRP).

In the first stage, 20 g of acrylamide (AM) is mixed with 100 g of distilled water. Following the dissolution of methylenebisacrylamide (MBA) in the solution, 0.6 percent Alginic Acid Sodium Salt from Brown Algae (Alg) is added to the solution. Since completely dissolving of Alg into the solution takes time, the solution was stirred for 24 h. The dissolved oxygen was removed the next day using argon, and APS was added to the solution. Gelation occurred by putting the molds comprising hydrogels in a 60 °C oven for three hours. This hydrogel is shortened and represented as H-a. The H-a pieces were ripped apart and put in a synthetic solution comprising 2.24 gr/lit lime, 29.82 lit/gr KCl, and 23.38 gr/lit distill water. Figure 7 shows the hydrogel granolas that increased in the presence of water. Figure 8 shows the in situ forming of the hydrogel by chemical cross-linking and an ionic interaction between the calcium ions and alginate. Figure 9 shows the in situ hydrogel forming using an enzymatic cross-linking reaction with H_2_O_2_ and horseradish peroxidase (HRP) [81].

#### 2.3.2. Hydrogels Containing Nanosilica Particles (NSi)

Acrylamide monomers (AM) were introduced to distilled water containing nanosilica at different concentrations of nanosilica/AM = 0.5%, 10%, 20%, and 50%. The solution was rapidly stirred after the cross-linker, N, N’-methylenebisacrylamide (MBA), and the initiator, ammonium persulfate, were added. The hydrogels were then dried at an 80 °C temperature. After drying, the hydrogels were crushed in a coffee grinder and sieved to create a powder with particle sizes ranging from 75 to 425 m. From this point on, these hydrogels are labeled with a number indicating the percentage of nanosilica particles, such as H-0, H-5, H-10, H-20, and H-50.

### 2.4. Water Glass Hydrogel (H-WG)

In a 3:7 ratio, sodium silicate was added to distilled water and stirred for 30 min. The solution was slightly neutralized with 14 percent (*wt*) sodium hydroxide prior to polymerization (NaOH). The solution was then placed for three hours at 60 °C. The hydrogel was then removed from the beaker, torn apart, and dried in an oven at 80 °C. After drying, the hydrogels were crushed in a coffee grinder and sieved to create a powder with particle sizes ranging from 75 to 425 m.

## 3. Hydrogel Absorption Test

**Figure 10:**
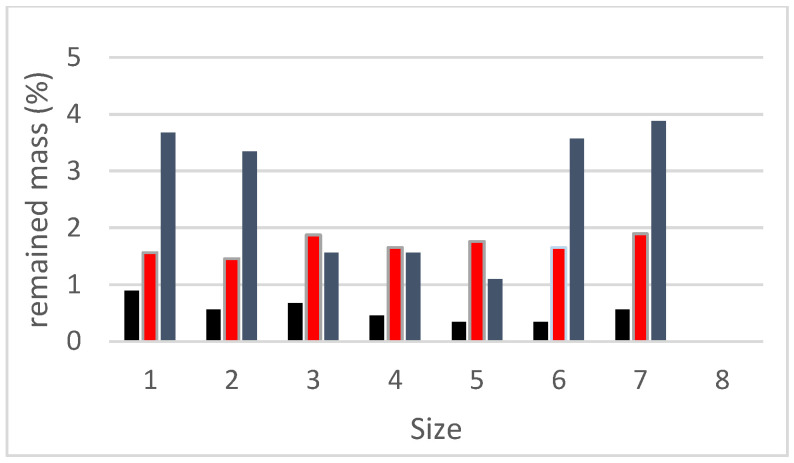
Dry hydrogel mass ratio retained on each sieve for the synthesized hydrogels.

**Figure 11:**
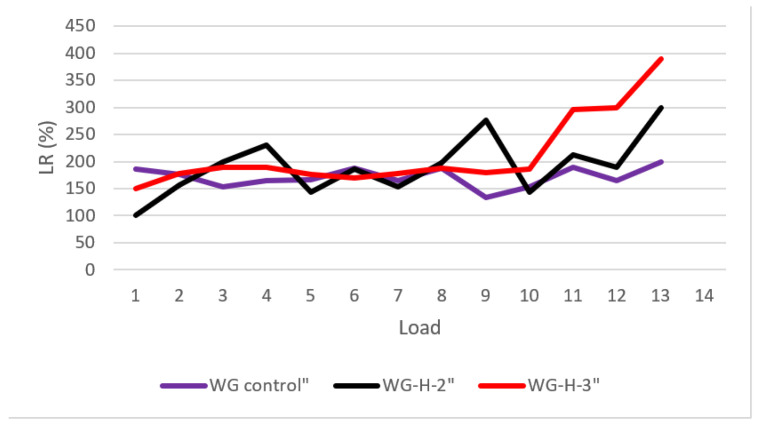
Load-regain percentage for the control and water glass pastes containing hydrogels.

**Figure 12:**
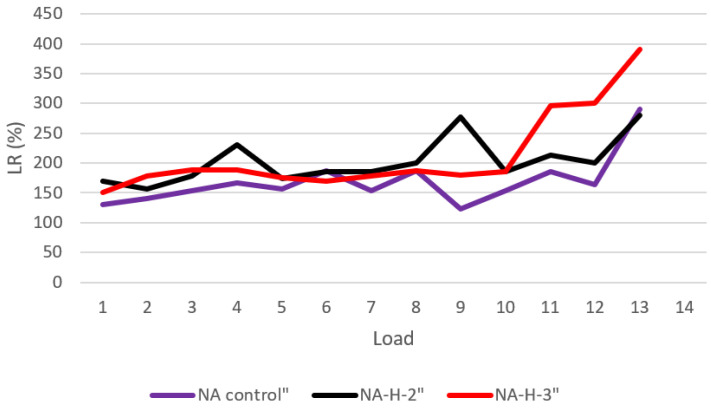
Load-regain percentage for the control and nanosilica particles (NSi) pastes containing hydrogels.

The teabag technique was used to test hydrogel absorption in artificial pore solutions of cement pastes. Each hydrogel was put into three teabags and immersed in the solutions for 0.1 g. The teabags were withdrawn from the solution at regular intervals, and the surfaces were gently dried with Kimwipes to eliminate any excess solution. Since some solution might become entrapped between the hydrogel particles or adsorbed onto the particle surface, no pressure was applied to the hydrogel throughout mass measurement and handling to avoid hydrogel damage. Subsequently, their mass was instantly determined on an analytical balance with a precision of 0.001 g, and their absorption was calculated utilizing Equation (1), and the average was given. Figure 10 shows Dry hydrogel mass ratio retained on each sieve for the hydrogels synthesized with three columns as H-2, H-3, H-a (see Section 2.3). Figure 11 shows the load-regain percentage for the control and water glass pastes containing hydrogels. Figure 12 shows the load-regain percentage for the control and nanosilica particles (NSi) pastes containing hydrogels.

### 3.1. Autogenous Shrinkage Test

**Figure 13:**
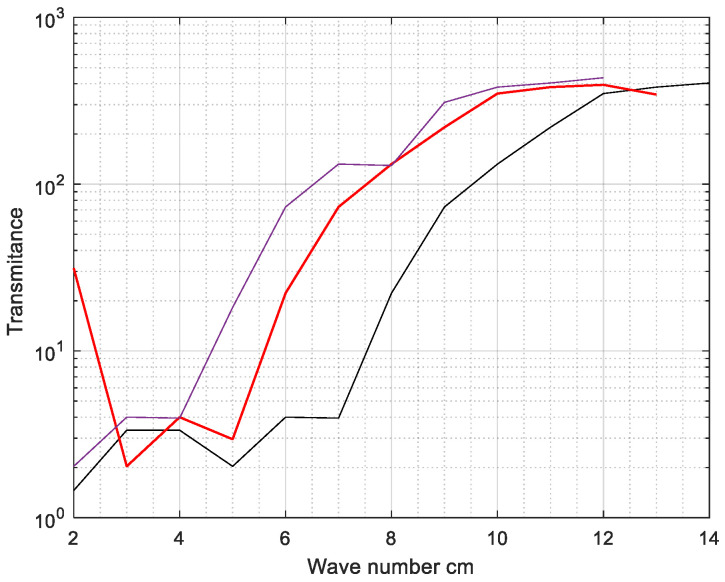
The shrinkage test analysis of cement clinker, nanosilica and water glass and healing product of the H-a hydrogel. Hydrogel swelling ratios as a function of time in cement pore solution are represented in (grey). Strain over time (autogenous shrinkage) for cement mortars with (out) hydrogels shown by (red). Water-to-cement (*w/c*) ratios are presented for each mortar specimen in addition to hydrogel composition (% AA) (violet).

The linear autogenous shrinkage of the cement samples was assessed in this investigation based on ASTM C1698-09. This approach successfully eliminates moisture loss and reduces the constraint to volume change while hardening. For each combination, three thin corrugated plastic tube samples with lengths and outer diameters of roughly 420 mm and 29 mm were made, and the average was reported. The specimen was put horizontally on corrugated plastic trays to reduce breakage and limit length change. The length of the samples was analyzed every 12 h for the first week. Measuring began at the final set point chosen by the Vicat needle test. All of the mixes were sufficiently workable to fill the corrugated tube within a few minutes of the completion of the mixing process. Figure 13 shows the shrinkage test analysis of cement clinker, Nano silica and water glass and healing product of H-a hydrogel. Here, Hydrogel swelling ratios as a function of time in cement pore solution are represented in (grey). Strain over time (autogenous shrinkage) for cement mortars with(out) hydrogels shown by (red). Water-to-cement (*w/c*) ratios are presented for each mortar specimen in addition to hydrogel composition (% AA) (blue). In this figure, hydrogel is designated by the letters H- 2, H-3, and H-a, consists of acrylic-based polymer hydrogels that do not contain silica component. As predicted, H-a had the lowest absorption relative to H-2 and H-3; this behavior was caused by the manufacturing procedure utilized for this hydrogel, which involved immersing the hydrogel in an ionic solution for around 24 h following gelation. Long-term exposure of H-a to the ionic solution enhanced cation diffusion into H-a and the creation of ionic crosslinks between the negative charges polymer network, both resulted in a decreased absorption capacity of H-a when immersed in the extracted pore solution.

### 3.2. Interaction of Hydrogels and Cementitious Materials and the Effect of Hydrogels on the Properties of Cementitious Materials

In this study, there was a significant difference in the behavior of several hydrogels in the extracted cement pore solution. This observation raises questions about the validity of hydrogel absorption assays that use solely the extracted pore solution to evaluate hydrogel absorption in cement paste. The influence of various chemical compositions of hydrogels on the electrical resistivity, hydration, strength, shrinkage, and autogenous nature of the cement paste was studied and addressed.

### 3.3. Compressive Strength

**Figure 14:**
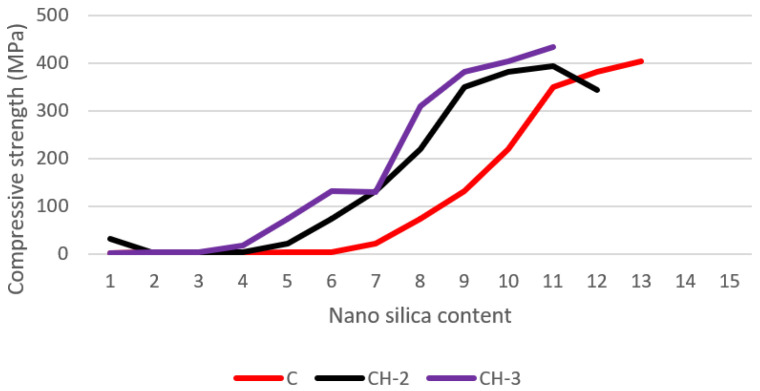
The compressive strength of nanosilica.

**Figure 15:**
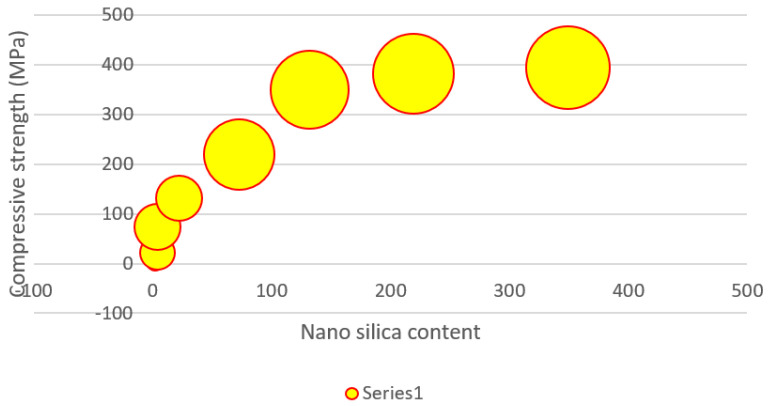
Atomic ratios of nanosilica cement paste and healing products formed in a slice of cement pastes.

**Figure 16:**
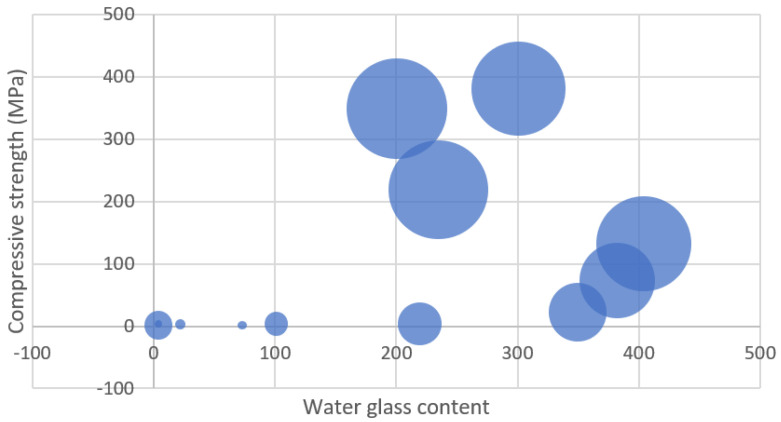
Atomic ratios of water glass cement paste and healing products formed in cement pastes.

Figure 14 depicts the compressive strength (CS) test results of C, C H-2, and C H-3 at 3, 7, and 28 days. The CS of all pastes raised with continued curing and time. The inclusion of hydrogels reduces CS because of the creation of macro gaps in the microstructure that function as stress concentration sites. The most significant drop in CS is evident in C H-2, which has significantly bigger macrovoids than C H-2 and C. While the overall *w/c* of C H-2 and C H-a was similar, there were minor differences in the CS of these two pastes. The fundamental cause of these differences is the variation in the size and quantity of macrovoids in these two pastes. C macrovoids were smaller in size but more numerous than C H-2 macrovoids. Figure 15 shows the atomic ratios of nanosilica cement paste and healing products formed in a slice of the cement pastes. Figure 16 shows the atomic ratios of Water glass cement paste and healing products formed in cement pastes. In this figure, the compressive strength of the control cement paste, C, including Nano-silica particle hydrogels, C H-0, C H-5, and C H-10, as well as the compressive strength of the cement pastes with colloidal silica and water glass. The compressive strength of the cement pastes containing hydrogels was found to be lower than that of the control cement paste.

### 3.4. Absorption Test

**Figure 17:**
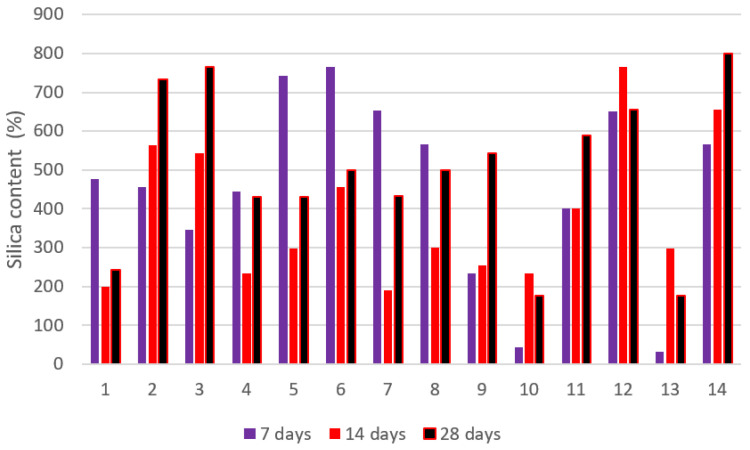
Nanosilica content of the slag cement pastes at 7, 14, and 28 days of age.

**Figure 18:**
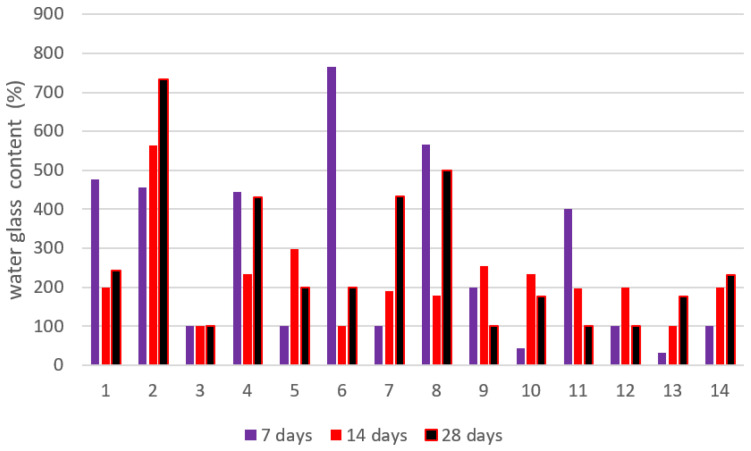
Water glass content of the slag cement pastes at 7, 14, and 28 days of age.

The higher pH of the synthetic pore solution in comparison to pure water might explain the higher absorption. It is worth noting that the influence of cations such as Na^+^, K^+^, and Ca^2+^ in the synthetic pore solution, which inhibits absorption via the screening impact and difficult formation, has not appeared to counter the absorption increment caused by pH change. The electrostatic interaction between negatively charged NSi and partly positively charged amide groups in the hydrogel network that constrains the expansion of the hydrogels might be one explanation for this phenomenon. In the synthetic pore solution, H-25 absorbed somewhat more than the other hydrogels in the early stages of absorption. Figure 17 presents the nanosilica content of the slag cement pastes at 7, 14, and 28 days of age, and Figure 18 shows the water glass content of the slag cement pastes at 7, 14, and 28 days of age.

## 4. Conclusions

The purpose of this study was to gain knowledge about the interaction between hydrogels of various chemical compositions and cementitious materials and the influence of the hydrogels on fostering self-healing behavior in cementitious pastes. This research provides a basic experimental investigation into the self-healing behavior of cementitious materials in the presence of hydrogels. All of the hydrogels employed in this study were synthesized in-house. Furthermore, a silica additive was added to some hydrogels. The hydrogels employed in this study were classified into two types. The first group contained hydrogels that did not have silica addition, whereas the second included hydrogels with a silica additive. Nanosilica particles and water glass were employed as silica additions. The influence of solid particles on hydrogel absorption behavior was described in this study. The internal curing behavior of hydrogels containing silica addition was investigated, and results about their healing effectiveness were derived. The self-healing behavior of cement pastes with and without hydrogels was explored because of the additional hydration of unhydrated cement particles.

Furthermore, the self-healing behavior of extra cementitious materials (slag, fly ash, and VCAS) was studied. The absorption value gained in the extracted pore solution through a teabag test might differ from the absorption estimation gained in the cement mixture. Chemical interactions between the hydrogel surface and hydrating cement particles were shown to significantly affect the hydrogel absorption in cement mixtures. Comparing the control paste, cement pastes containing hydrogels indicated less autogenous shrinkage. The influence of hydrogels on autogenous shrinkage was found to be chemically dependent; the hydrogel with a delayed desorption ratio displayed very low shrinkage in the cement matrix. The inclusion of hydrogels has shown a general decrease in the CS and electrical resistivity of cement material—such decreases are caused by the creation of macrovoids.

## Figures and Tables

**Table 1:** Compositions of the hydrogels used in the experiment.

NO.	Hydrogel	Distilled Water	AA (%)	AM (%)	NaOH (%)	MBA (%)	Alg (%)	APS (%)	NSi Powder (gr)	Colloidal NSi (gr)	Water Glass (gr)
1	H-2	100	10	10	1.35	0.05	-	0.128	-	-	-
2	H-3	100	2	18	0.27	0.05	-	0.128	-	-	-
3	H-a	100	-	20	-	0.05	0.6	0.128	-	-	-
4	0%- Reference Hydrogel	100	-	20	-	0.05	-	0.64	-	-	-
5	5%-NSi	100	-	20	-	0.04	-	0.54	1	-	-
6	15%-NSi	100	-	20	-	0.04	-	0.54	2	-	-
7	25%-NSi	100	-	20	-	0.04	-	0.54	4	-	-
8	40%-NSi	100	-	20	-	0.04	-	0.54	10	-	-
9	CNSi	50	-	20	-	0.04	-	0.54	-	50	-
10	WG	70	-	20	14	0.04	-	0.54	-	-	30

## Data Availability

Not applicable.
